# Localization of Immersed Sources by Modified Convolutional Neural Network: Application to a Deep-Sea Experiment

**DOI:** 10.3390/s21093109

**Published:** 2021-04-29

**Authors:** Xu Xiao, Wenbo Wang, Lin Su, Xinyi Guo, Li Ma, Qunyan Ren

**Affiliations:** 1 Institute of Acoustics, Chinese Academy of Sciences, Beijing 100190, China; xiaoxu@mail.ioa.ac.cn (X.X.); wangwenbo215@mails.ucas.ac.cn (W.W.); sulin807@mail.ioa.ac.cn (L.S.); guoxinyi@mail.ioa.ac.cn (X.G.); mary1968@tom.com (L.M.); 2 Key Laboratory of Underwater Acoustics Environment, Chinese Academy of Sciences, Beijing 100190, China; 3 University of Chinese Academy of Sciences, Beijing 100049, China

**Keywords:** vertical linear array, Gauss regression output, source ranging, convolutional neural

## Abstract

A modified convolutional neural network (CNN) is proposed to enhance the reliability of source ranging based on acoustic field data received by a vertical array. Compared to the traditional method, the output layer is modified by outputting Gauss regression sequences, expressed using a Gaussian probability distribution form centered on the actual distance. The processed results of deep-sea experimental data confirmed that the ranging performance of the CNN with a Gauss regression output was better than that using single regression and classification outputs. The mean relative error between the predicted distance and the actual value was ~2.77%, and the positioning accuracy with 10% and 5% error was 99.56% and 90.14%, respectively.

## 1. Introduction

Source localization is a long-term research topic in underwater acoustics, and approaches based on matched field processing (MFP) are widely applied [1]. The concept of MFP for source localization relies on determining the position of the best match between real data and replicas generated at a certain grid but limited by some common problems, such as environment mismatch and the convergence difficulty in the optimization algorithm [2]. Recently, machine learning (ML), including deep-learning methods, such as those involving feedforward neural networks (FNNs) and convolutional neural networks (CNNs), has become a research hotspot owing to the high performance of these methods for classification and feature extraction tasks. In the field of image recognition, AlexNet [3] was proposed in the ImageNet large-scale visual recognition challenge benchmark, confirming the dominant position of CNNs in the field of computer vision. Hinton et al. [4] introduced an FNN into speech recognition, driving the rapid growth of speech recognition technology. In source localization, ML is a data-based approach that exploits the function between underlying training data sets and corresponding source ranges and then performs source localization on testing data sets [5]. Various studies have found that ML achieved lower error in source ranging than MFP, especially in complex ocean environments with low SNR [5,6,7]. In addition, progressively increasing network architectures, such as FNN [5,6], TDNN [7,8], CNN [9,10], ResNet [11,12,13], and Inceptions [10], are applied to underwater acoustic localization to mine deep features and decouple sound source information from the underwater acoustic environment. This application of ML to source ranging has been demonstrated to achieve good localization results in both synthetic and experimental data sets collected on the sea. However, in the published literature, the outputs of ML based on CNNs are extremely scattered within estimated ranges, especially when the SNR is low, although they do provide stable trends. These scattered estimation results can be confusing in terms of physical interpretation and practical applications, especially when encountering a very limited data set size.

In our recent study [14], a Gaussian distribution for output layer labels used in CNN transfer learning to express the uncertainty of ranging estimation and achieved good ranging results on sea trial data. However, no systematic elucidation and analysis for the underlying mechanism was given in that paper. Based on our previous results, this paper further investigates the benefit of changing the conventional format of CNNs as a Gaussian distribution for output layer labels. Considering the errors between the “true” distances from a source to a receiver, as calculated from GPS points, the standard deviation of the Gaussian distribution label reduced the divergence of the estimated results. The CNN source-range performance for at-sea data compared with that of CNN regression layer and classification layer outputs were significantly improved. In addition, the performance of the modified CNN with different standard deviations was examined for real data with different signal-to-noise ratios (SNRs).

This paper is organized as follows: Section 2 describes the details of the at-sea experiment, the modified CNN framework for source ranging is presented in Section 3; the real-data processing is given in Section 4; the results and analyses are given in Section 5, and Section 6 concludes the paper.

## 2. Description of the Experiment

**Figure 1:**
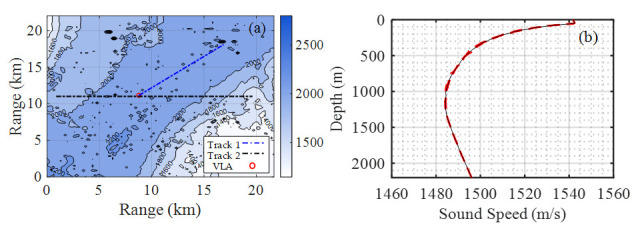
(**a**) Bathymetry and survey lines in the experimental area as also presented in [14]. The contours represent the water depths in this area. The dashed blue line represents track 1, the dashed black line (horizontal) represents track 2, and the red circle (at the intersection of dashed blue and black lines) represents the position of the vertical line array. (**b**) Sound speed profiles measured in situ using conductivity, temperature, and depth (CTD). The red line represents the CTD measurements, and the black line is the average.

To study sound source ranging, sea trials were carried out in the South China Sea in 2017. The experimental data in this paper adopted the data used in the literature [14]. In this experiment, the sound source was towed by a source ship with pre-designed routes, as indicated by the blue and black dashed lines in Figure 1a, while the recording system was an 8-element vertical line array (VLA) moored on the seafloor, which recorded the sound data of different source distance. The bathymetry of the experimental area measured using a multibeam sonar is shown in Figure 1a.

The top element of the VLA was located at a depth of 1880 m, the bottom sensor of the VLA was fixed 20 m from the seabed, and the spacing between the elements was 20 m. According to the depth sensor recordings, the VLA remained straight throughout the entire experiment. As shown in Figure 1a, the trajectory of the source ship can be divided into two main lines, track 1 and track 2. The bathymetry of track 1 (denoted by the blue dashed line) was relatively flat, whose farthest distance to the VLA is about 10 km. The bathymetry of track 2 (denoted by the black dashed line) was moderately range dependent along latitude and had a slope of about 9.65°. In track 2, the source ship repeatedly sailed to the east and west of the VLA, reaching a maximum of approximately 8 km west and approximately 12 km east of the VLA.

Figure 1b shows the sound speed profiles (SSPs) calculated from in situ measured conductivity, temperature, and depth (CTD) before, after, and during the source, the ship runs. The source ship speed was approximately 4 knots, and an acoustic transducer was towed from the ship with varying depths between 50 and 100 m, which were highly correlated due to the status of the source ship (i.e., maneuvering, etc.).

To test the ranging effect under a low SNR, A spectral signal with multiple narrow bands (narrowband frequencies of 63, 79, 105, 126, 160, 203, 260, and 315 Hz) was transmitted at different SNRs. Each group of signals contained four segments, denoted E1, E2, E3, and E4. The SNR was sequentially reduced by 0, 5, 10, and 15 dB for 60 s durations at 10 s intervals. The experimental data were recorded using an 8-element VLA fixed 20 m above the seabed.

## 3. CNNs and the Working Framework

### 3.1. Classic CNNs for Source Localization

For source ranging, CNNs are built to learn the mapping relationship between the sound data and the source distance. Applications using CNNs for underwater acoustics [9,10] take advantage of the ability of CNNs to exploit the correspondence between sound field features and source location through training. Most CNN-based source localization methods can be roughly separated into regression tasks and classification tasks. Normally, source ranging is initially modeled as a classification task, which needs to balance the density of the classifier categories and the sparsity of the data for the accuracy of the source ranging. Recently, source ranging has been preferentially modeled as a regression task for more robust performance, particularly for moving sources.

**Figure 2:**
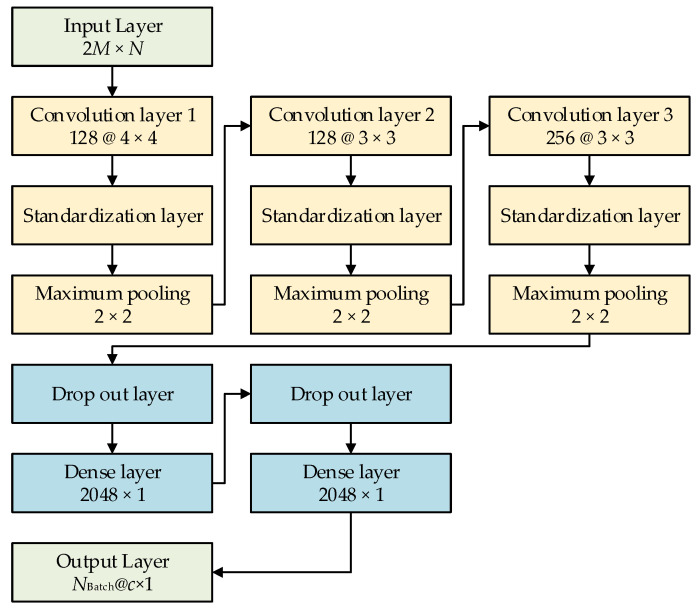
Schematic of a typical convolutional neural network.

Figure 2 shows the schematic of a typical CNN, as adopted in this study. Generally, the complex spectrum of sound data contains a large amount of target information and waveguide information for source ranging. For a vertical array recording system with *N* elements, if *M* discrete frequency acoustic data are used, then the size of the input layer is a matrix of 2*M* × *N*, in which 2 refers to using both the real and imaginary parts of the complex spectrum, as calculated from the sound data. Convolution layer 1 was composed of 128 convolutions 4 × 4-sized cores, which were connected to the standardization layer and the maximum pooling layer with a step size of 2 × 2; convolution layer 2 was composed of 128 convolution 3 × 3-sized cores, which were connected to the standardization layer and the maximum pooling layer with a step size of 2 × 2; and convolution layer 3 was composed of 256 convolutions 3 × 3-sized cores, which were also connected to the standardization layer. A dropout layer with a coefficient of 0.3 was introduced between the convolution and the full connection layers. A fully connected layer, with 2 layers of neurons, comprised a 2048-neuron fully connected neural network. A dropout layer with a coefficient of 0.5 was introduced between the full connection and the output layers. By doing several tests on underwater acoustic data, this CNN model was proved to have high reliability and convergence.

This study analyzed three types of output layers: a regression layer with a single output value, a Softmax layer for distance classification, and a regression layer with Gaussian distribution output. The results from these three types of output layers were compared in this paper.

In practice, for neural networks, several steps are adopted to obtain a better generalization of actual acoustic field data and prevent overfitting from occurring in the training process; for example, creating more acoustic field data, selection of the neural network model (including the number of neurons and hidden layers) following the data complexity, regularization of network parameters [15], and addition of a dropout layer [16] in the network training.

### 3.2. Modified CNNs with a Gauss-Regression Layer

In most CNN-based source-ranging methods, the CNN outputs are often set as *N* discrete points. Generally, the max receiving range is separated into *N* points, with a fixed interval of Δ*r*. The corresponding CNN outputs can then be described as follows:

(1) r=[Δr,2Δr,3Δr,⋯,NΔr].

In practice, these settings can invoke unexpected estimation errors when the labeled distance is biased by true distance. In general, there is a certain distance between the onboard GPS and the towed sound source, which will change with different ship sizes and towing conditions of the sound source. Thus, the estimated range usually diverges from the true value.

In an attempt to increase the precision of traditional CNN methods and consider the actual range bias with that of GPS calculated distance, the label for the CNN output layer at measured distance *r*_GPS_ was designed as follows:

(2) $$L_{\mathrm{Gauss}}=\left[e^{-\frac{{\left(\Delta r-r_{\mathrm{GPS}}\right)}^{2}}{2\sigma^{2}}},e^{-\frac{{\left(2\Delta r-r_{\mathrm{GPS}}\right)}^{2}}{2\sigma^{2}}},\cdots ,e^{-\frac{{\left(N\Delta r-r_{\mathrm{GPS}}\right)}^{2}}{2\sigma^{2}}}\right],$$

where *σ* describes the possible error between the real distance with the value as obtained from the GPS, and **L**_Gauss_ is the new label for the CNN output, which has the same vector size of *N* × 1 as those of traditional CNN approaches.

The training process for the modified CNN was the same as that used by classical CNN approaches. The testing process was as follows: the modified CNN outputted an *N* × 1 string when a test data set was fed in. As the position *t*_position_ of the max value in the string could be easily found, the source range provided by the proposed method could be expressed as follows:

*r*_estimate_ = *t*_position_ Δ*r*. (3)

The output sequence of the Gauss regression layer was in the form of a Gaussian-probability density function with a mean value equal to the source range. A training data set can be simulation data, experimental data, or a combination of both where the simulation data can compensate for the shortage of real data. The experimental data can alleviate the problem of the excessive error caused by the mismatch between the model and the parameters. In this study, only the simulation data were used as replicas, and the predicted parameters only included the source range, not the source depth.

### 3.3. CNN Processing

**Figure 3:**
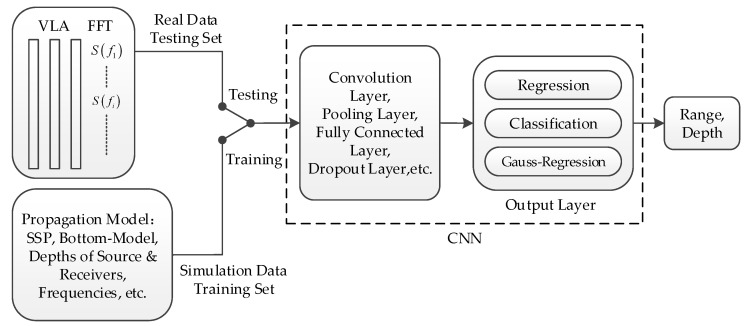
Process diagram of the convolutional neural network (CNN) for processing field sound data received by a vertical array. The real data were set as the test set, and the simulated data were set as the training set. The CNN was trained to output the corresponding Gaussian label of the training set, and the target was then positioned by the trained CNN on the test set to verify the performance of the model.

Figure 3 shows a diagram of the CNN used to locate the source by processing the field sound data received by a vertical array. The output layer of the CNN was divided into three layers: regression, classification, and Gauss regression. The regression layer pertained to returning a continuous output value as the ranging results. The classification layer pertained to dividing the output range into grid form, setting the true value to 1 and the rest to 0.

## 4. Data Simulation and Preprocessing

To expand the dataset, environmental parameters and spatial distribution were used to produce replica datasets to compensate for the lack of real data at certain distances. Using either prior information or in situ measured environmental parameters and geometrical and source settings, such as SSPs, a seabed model and parameters, the depths of sources and hydrophones, and signal frequencies, a sound field replica data set as recorded by the VLA can be calculated via a sound field calculation code, herein, Kraken-C [17]. Before inputting into the CNN, the data were first normalized by procedures presented by Niu et al. [6], and a net was used to locate the source by analyzing the data set as provided by the VLA.

**Figure 4:**
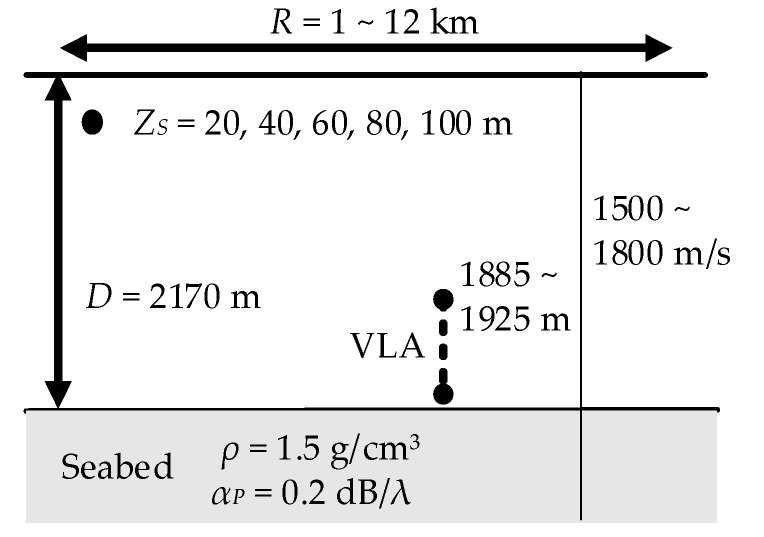
Synthetic ocean environment model, where R represents the range between source and receiver; ZS represents the depth of source; D represents the depth of the sea; ρ and αP represent the density and attenuation coefficient of the seabed, respectively.

The simulation was carried out using the Kraken-C sound field calculation program. The SSPs used in the simulation, three measured ones and one average one, were used to obtain a smooth sound field structure. The water depth at the VLA was approximately 2170 m, and the seabed was regarded as a single-layer seabed. The density of the seabed was set at 1.5 g/cm^3^, and the attenuation coefficient was set at 0.2 dB/wavelength. Because the sound speed of seabed has considerable influence on the sound field, it was varied from 1500 to 1800 m/s at intervals of 50 m/s. The source depths were sequentially set at 20, 40, 60, 80, and 100 m. Considering the placement error of the VLA depth, the first element of the VLA was 1885, 1905, and 1925 m, respectively. The horizontal distance interval was 0.02 km, and the longest distance was 12 km. A total of 252,420 (4 SSPs × 7 bottom speeds × 5 source depths × 601 ranges × 3 VLA depths) copies of sound field data sets were generated by employing the above-mentioned parameters and used as a training dataset. 10% percent of the training set was used as the validation set for diagnosis during DNN training. A diagram of the synthetic ocean environment is shown in Figure 4.

A CNN with different output modes was trained using simulation data, and the network was optimized by Adam’s adaptive optimization algorithm [18]. A total of 10,000 epochs were trained with an initial learning rate of 1 × 10^−4^ and a regularization factor of 5 × 10^−4^ to ensure adequate network training. For the receiving VLA sound field data, fast Fourier transform was performed on the signal of each element, with a time window of 1 s and step size of 0.25 s. Further, each signal was normalized by the following formula:

(4) $$P^{\prime }\left(f,z_{n}\right)=\frac{P\left(f,z_{n}\right)}{\sqrt{\sum_{n=1}^{N}{\left|P\left(f,z_{n}\right)\right|}^{2}}},$$

where *f* represents the frequency and *z_n_* represents the depth. These feature vectors contained abundant information on the properties of the sound field and the source, which was extracted and mapped to the target location by the CNN architecture. The preprocessing results were inputted into three types of CNNs. The classification output layer divided the output into different categories with a spacing of 0.02 km for training. The regression output layer was the output in the form of the continuous distance value of the source. The Gauss regression output layer incorporated a Gaussian distribution using a one-dimensional Gaussian function with a standard deviation of 0.1 and a mesh spacing of 0.02 km. Tracks 1 and 2 contained 3371 and 14,400 data sample points, respectively, for each SNR. The statistical data described in the following section corresponds to all the data under each SNR.

## 5. Results and Analyses

To quantify the performance of the ranging method, the probability of credible localization [19] (PCL) and the mean absolute percentage error (MAPE) were used as the criteria for the performance tests. The PCL was used to estimate the reliability of the ranging results by indicating that the relative ranging error estimated by the method was less than the percentage of the given value at all test sample points as follows:

(5) $$P_{\mathrm{PCL}-\lambda \%}=\frac{\sum_{k=1}^{K}\eta (k)}{K},\;\eta (k)=\left\{ \begin{array}{l} 1,\;\frac{\left|{\tilde{r}}_{0}(k)-r_{0}\right|}{r_{0}}\times 100\%\leq \lambda \%\\ 0,\;\mathrm{else}, \end{array}\right.$$

where *K* is the sample size of the test data, ${\tilde{r}}_{0}$ is the estimated range, and *r*_0_ is the GPS range. Equation (5) shows that when the relative error of the distance estimate is less than *λ*%, the estimated distance is considered to be reliable, and the PCL −*λ*% is the percentage of the number of reliable ranging in the total number of samples. PCL −*λ*% is a criterion that is very strict with the convergence of predictions but tolerates errors within a certain range. Even if the prediction has high accuracy, when the prediction is unstable and divergent, the PCL −*λ*% can decrease sharply.

The MAPE was defined to measure the difference between the estimated and real value data, representing the percentage of the mean relative error of the estimated distance at all test sample points as follows:

(6) $$\mathrm{MAPE}\;=\;\frac{100\%}{n}\sum_{i=1}^{n}\left|\frac{{\hat{y}}_{i}-y_{i}}{y_{i}}\right|,$$

where *i* = 1, 2, 3, …, *n* represents the numbers of output nodes and $y_{i}$ and ${\hat{y}}_{i}$ represent the true label and the estimated value of each output node, respectively.

**Figure 5:**
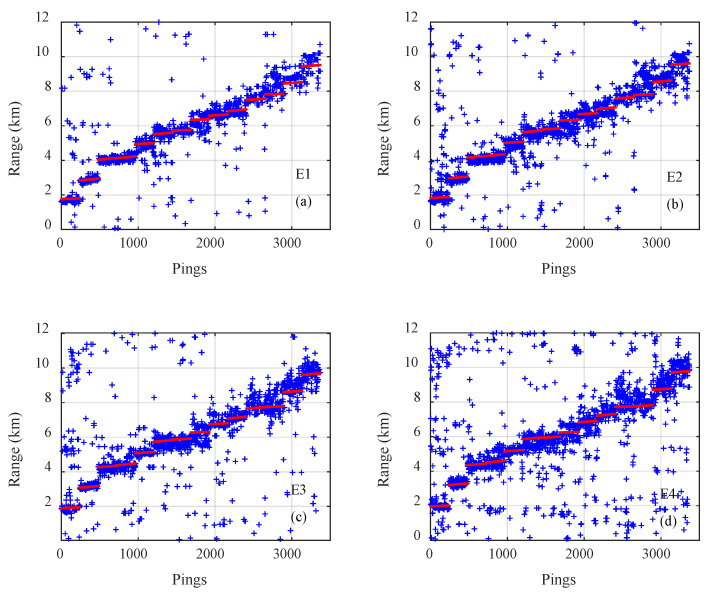
The true range (red) and the estimated range of the classification convolutional neural network (blue) for all 3371 samples on track 1 with emission energy of (**a**) E1, (**b**) E2, (**c**) E3, (**d**) E4.

**Figure 6:**
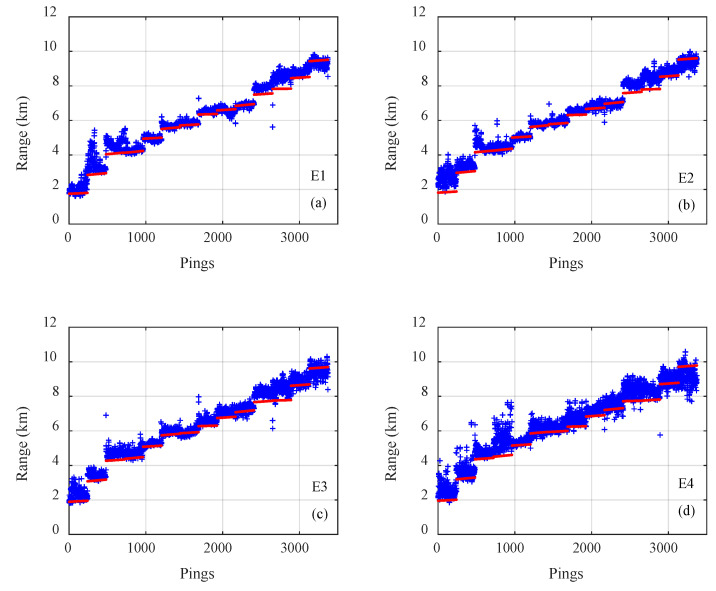
The true range (red) and the estimated range of the regression convolutional neural network (blue) for all 3371 samples on track 1 with emission energy of (**a**) E1, (**b**) E2, (**c**) E3, (**d**) E4.

**Figure 7:**
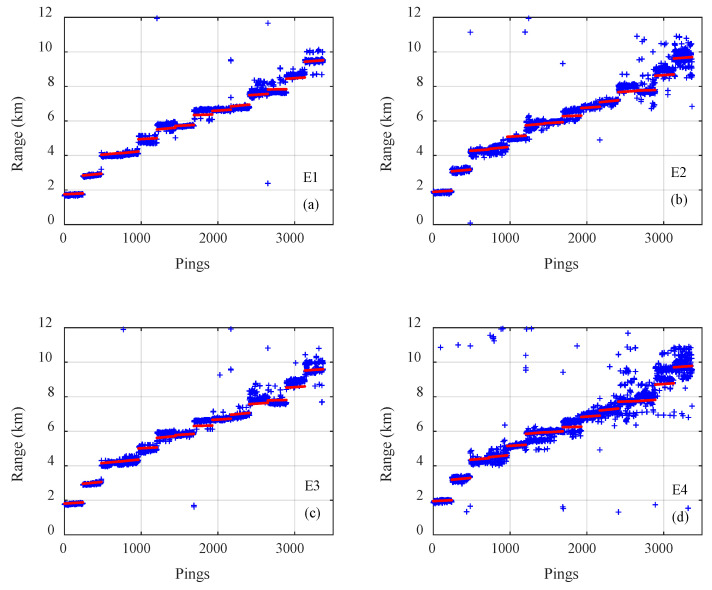
The true range (red) and the estimated range of the Gauss regression convolutional neural network (blue) for all 3371 samples on track 1 with emission energy of (**a**) E1, (**b**) E2, (**c**) E3, (**d**) E4.

The results of on the three types of CNNs for all 3371 samples on track 1 are presented in Figure 5, Figure 6 and Figure 7, where the pings are the numbers of the samples in the test dataset, the estimated distances of samples are given by blue plus signs and true distances given by red dots. The outputs of the classification CNN and the regression CNN were scattered and diverged from the true values, as shown in Figure 5 and Figure 6. Under the same conditions, the Gauss regression CNN showed stronger convergence ability and ranging performance, particularly for the close and middle range, as shown in Figure 7.

**Figure 8:**
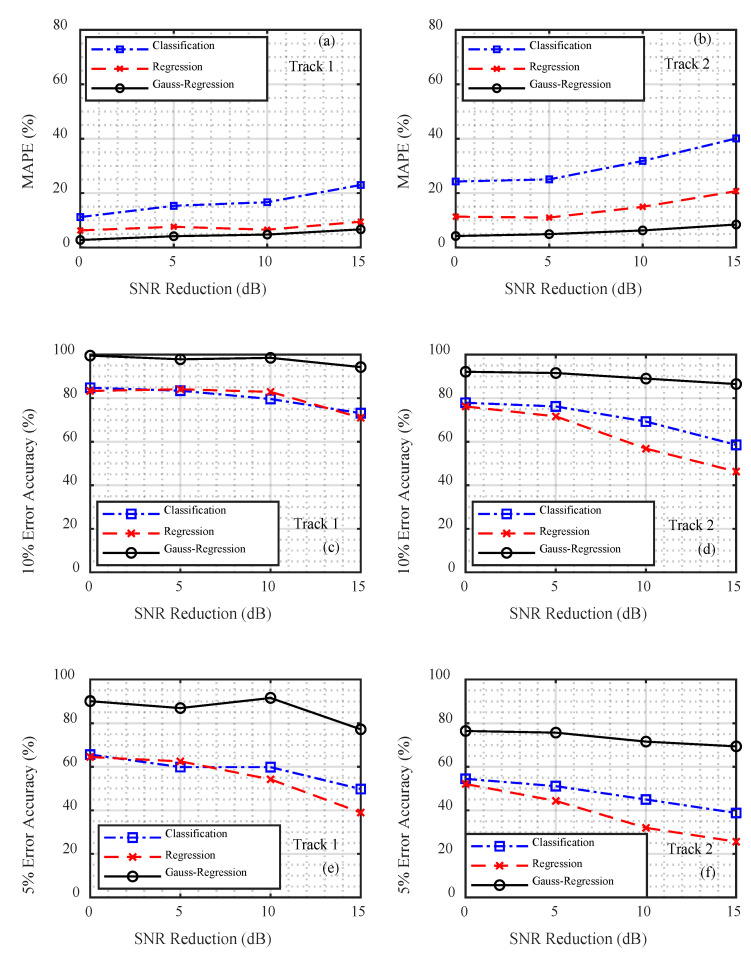
Comparison of the track 1 and track 2 data ranging results for the different convolutional neural networks. (**a**,**b**) Mean absolute percentage error (MAPE), (**c**,**d**) probability of credible localization (PCL) −10%, and (**e**,**f**) PCL −5%.

When comparing different output layers, the network architecture, network parameters, and training mode were the same. The following conclusions can be drawn from the comparison chart presented in Figure 8. The Gauss regression CNN demonstrated the best performance in terms of average relative error and accuracy. When the emission energy was 0 dB, the PCL −5% and PCL −10% of the Gauss regression CNN reached accuracies of 99.56% and 90.14%, respectively, which were higher than those of the classification CNN (84.81% and 65.62%) and the regression CNN (83.33% and 64.49%). With a decrease in SNR, the ranging accuracy of the three methods decreased, but the degree of degradation of the Gauss regression CNN was smaller than that of the other two methods. In track 1, for example, when the relative SNR was reduced from 0 dB to −15 dB, the Gauss regression CNN curve displayed a drop of 5.27% and 12.93% within the error range of 10% and 5%, respectively, with drop rates of 11.69% and 15.84% for the classification method and 12.31% and 25.51% for the regression method, respectively. Although the Gauss regression CNN showed better performance in the criterion of PCL, its MAPE was similar to the traditional regression CNN, while its PCL was much higher, which indicated its limitations in local searching ability and precision of estimation results.

The MAPE for the classification CNN was the highest; however, its 5% and 10% error accuracy was greater than that of the regression CNN. This was because the outputs of the different distances could not be effectively distinguished in the classification problem, while the output of the regression problem was a continuous result: the closer the distance to the real one, the smaller the error.

The MAPE of the regression CNN with a single distance in the output layer was lower than that of the classification CNN; however, its 5% and 10% error accuracy was the lowest. This showed that the regression method fully reflected the regression fitting ability of the deep neural network, and the relative average error of the output was very small. However, the corresponding positioning accuracy was not remarkably high, which may have been caused by a mismatch between the actual environment and the simulation model.

The MAPE and ranging accuracy of track 1 were less than those for track 2. Because the simulation environment used in this study was horizontally uniform and the seabed in track 1 was relatively flat, the relationship between the actual acoustic field characteristics and the simulation data was more consistent. The farthest distance between the two ends of track 2 pertained to an uphill environment. The simulation carried out in this study could not accurately simulate the acoustic field characteristics of the seabed as it fluctuated with distance, and the accuracy of the location was thus decreased.

**Figure 9:**
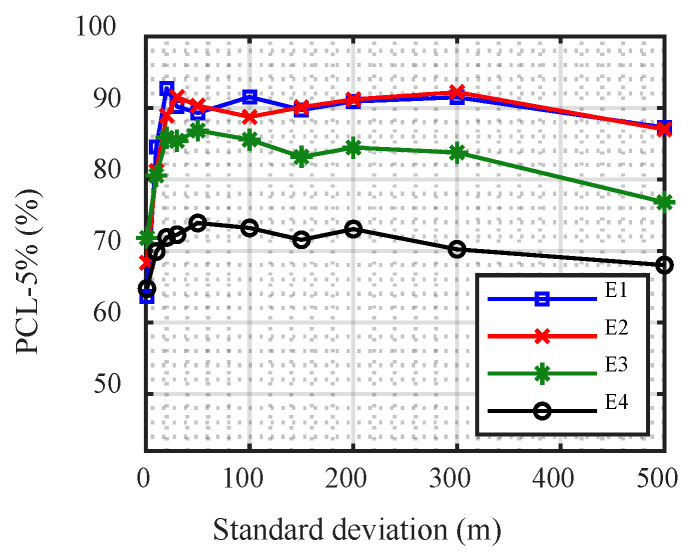
Performance of the modified convolutional neural network with different standard deviations.

The modified CNN with different standard deviations of the Gauss regression layer was compared to the different SNRs (Figure 9). For each SNR, at first, the PCL −5% rapidly increased with increasing standard deviation; however, it tended to stabilize. When the standard deviation reached a certain value, the improved performance of the CNN slowly declined. Moreover, the value of the standard deviation when the performance began to degrade was smaller with lower SNR.

## 6. Conclusions

Using simulated replicas as the training data set, the source distance was obtained from the multiple-narrow-band data received by a deep-sea vertical array using a trained CNN. The results showed that setting the output layer of the CNN in the form of a Gaussian distribution regression significantly improved the ranging accuracy and convergence ability. In addition, the Gauss regression CNN was least affected by the decrease in SNR compared with the traditional CNNs. However, the Gauss regression CNN showed a limitation in local searching ability and precision of estimation results.

When compares the two traditional CNNs, the results indicate that the ranging error of different estimated distances could not be effectively distinguished in the classification CNN, while the output of the regression CNN was a continuous result by minimizing the error between the true and estimated range, which leading to a higher MAPE for classification CNN, but a higher accuracy for regression CNN.

The modified CNN is more suitable for practical environments: the position in the classification problem pertained to different types of position relations, while the fuzzy change in the Gaussian function with distance was similar to the horizontal correlation of the actual sound field, which makes the model fully trained and a better fit. However, for complex oceanographic environments, the results showed that the CNN performance decreases significantly when the marine environment changes greatly with the distance, calling for further research on the environmental adaptation for source-ranging CNNs.

## Data Availability

Not applicable.
